# Extracellular-superoxide dismutase DNA methylation promotes oxidative stress in homocysteine-induced atherosclerosis: EC-SOD DNA methylation regulates atherosclerosis in *ApoE* ^–/–^ mice

**DOI:** 10.3724/abbs.2022093

**Published:** 2022-07-22

**Authors:** Shengchao Ma, Guanjun Lu, Qing Zhang, Ning Ding, Yuzhen Jie, Hui Zhang, Lingbo Xu, Lin Xie, Xiaoling Yang, Huiping Zhang, Yideng Jiang

**Affiliations:** 1 NHC Key Laboratory of Metabolic Cardiovascular Diseases Research Ningxia Medical University Yinchuan 750004 China; 2 Ningxia Key Laboratory of Vascular Injury and Repair Research Ningxia Medical University Yinchuan 750004 China; 3 The School of Basic Medical Sciences Ningxia Medical University Yinchuan 750004 China; 4 Department of Urology Clinical School of Medicine Ningxia Medical University Yinchuan 750004 China; 5 Departments of Prenatal Diagnosis Maternal and Child health Hospital of Hunan Province Changsha 410008 China

**Keywords:** homocysteine, oxidative stress, extracellular-superoxide dismutase, DNA methylation

## Abstract

In the present study, we investigate the effect of homocysteine (Hcy) on extracellular-superoxide dismutase (EC-SOD) DNA methylation in the aorta of mice, and explore the underlying mechanism in macrophages, trying to identify the key targets of Hcy-induced EC-SOD methylation changes. *ApoE* ^–/–^ mice are fed different diets for 15 weeks, EC-SOD and DNA methyltransferase 1 (DNMT1) expression levels are detected by RT-PCR and western blot analysis. EC-SOD methylation levels are assessed by ntMS-PCR. After EC-SOD overexpression or knockdown in macrophages, following the transfection of macrophages with pEGFP-N1-DNMT1, the methylation levels of EC-SOD are detected. Our data show that the concentrations of Hcy and the area of atherogenic lesions are significantly increased in *ApoE* ^–/–^ mice fed with a high-methionine diet, and have a positive correlation with the levels of superoxide anions, which indicates that Hcy-activated superoxide anions enhance the development of atherogenic lesions. EC-SOD expression is suppressed by Hcy, and the content of superoxide anion is increased when EC-SOD is silenced by RNAi in macrophages, suggesting that EC-SOD plays a major part in oxidative stress induced by Hcy. Furthermore, the promoter activity of EC-SOD is increased following transfection with the –1/–1100 fragment, and EC-SOD methylation level is significantly suppressed by Hcy, and more significantly decreased upon DNMT1 overexpression. In conclusion, Hcy may alter the DNA methylation status and DNMT1 acts as the essential enzyme in the methyl transfer process to disturb the status of EC-SOD DNA methylation, leading to decreased expression of EC-SOD and increased oxidative stress and atherosclerosis.

## Introduction

Increased production of superoxide anions leads to oxidative stress, which has been implicated in a number of cardiovascular diseases, including atherosclerosis (AS), hypercholesterolemia, hypertension, diabetic cardiomyopathy, diabetic retinopathy and ischemic heart disease [1]. Epidemiological evidence indicates that homocysteine (Hcy) is an independent risk factor for the development of AS [2]. Previous studies have demonstrated that Hcy may cause an imbalance between oxidation and oxidation resistance [ 3, 4] . However, it is not clear whether the development of AS is influenced by the association between Hcy and oxidative stress.

Oxidative stress is caused by an imbalance between the production of reactive oxygen and the body’s ability to detoxify the reactive intermediates and repair the resulting damage [3]. Superoxide dismutases (SODs) are a major cellular defense against oxygen free radicals and the formation of peroxynitrite [5]. All mammalian tissues contain three forms of SODs: cytosolic (Cu/Zn-SOD), mitochondrial (Mn-SOD) and extracellular (EC-SOD) [6]. EC-SOD, a copper- and zinc-containing glycoprotein secreted from vascular smooth muscle cells, is the most abundant SOD in vascular tissues; however, in atherosclerotic vessels, EC-SOD is also generated by lipid-laden macrophages [7]. Previous studies have indicated that EC-SOD is an important mediator for modulating vascular tone and inhibiting atherogenesis [ 8, 9] . High cholesterol level, high frequency of AS lesions, and high interstitial concentrations of superoxide anions have been reported in EC-SOD-null mice [10]. The mechanisms of oxidative stress-induced AS have been extensively studied [11]. However, the mechanisms of the association between oxidative stress and Hcy remain unclear. Hwang *et al*. [12] reported that Hcy promotes oxidative stress by downregulating the expression of EC-SOD in patients with AS. However, to the best of our knowledge there is no direct evidence explaining the mechanisms by which Hcy affects the expression of EC-SOD and disturbs the oxidation balance in oxidative stress-induced AS.

Epidemiological studies have demonstrated that a mildly elevated plasma Hcy level is an independent risk factor for the development of AS [13]. Hcy is a non-protein, sulfur-containing amino acid, which is formed exclusively by the demethylation of methionine [14]. Hcy is a precursor of S-adenosylmethionine (SAM), the primary methyl group donor for most biological methylations, particularly DNA methylation which is a form of epigenetic gene regulation that functions in conjunction with the altered binding profiles of transcription factors [15]. Together, these modifications often lead to the suppression of gene expression when they occur in regulatory genomic regions, particularly in the promoter regions [16]. It was reported that the fatty acid-binding protein gene became hypomethylated and the expression of mRNA was increased in cultured macrophages and foam cells treated with Hcy [17]. In the cycle of a methyl transfer reaction, Hcy is remethylated to methionine, then S-adenosylhomocysteine (SAH) is released as a by-product, and this cycle is maintained by DNA methyltransferases (DNMTs), including DNMT3 and DNMT1 [18]. The majority of research in DNA methylation is from developmental biology, cancer biology and studies with targeted deletions of mouse DNMT1 which is the ‘maintenance’ methyltransferase that ensures the correct transmission of the methylation profile from maternal to daughter cells during cell division [19]. In recent years, researchers have focused on the role of DNA methylation in AS, trying to provide novel insights into the cause of AS and better understandings of the epigenetic mechanisms underlying AS pathogenesis [20]. Laukkanen *et al*. [21] investigated the methylation of CpG islands located on the coding sequence of the EC-SOD gene in the AS lesions of hyperlipidemic rabbits. However, the association between DNA methylation and EC-SOD expression has not been elucidated in detail, particularly the mechanisms by which DNA methylation regulates the expression of EC-SOD and disturbs the balance between oxidation and anti-oxidation in the development of AS.

In the present study, we revealed the role and epigenetic regulation mechanism of EC-SOD on the effects of oxidative stress induced by Hcy. Our results indicated that DNMT1 mediates EC-SOD promoter hypomethylation to suppress its expression, and thus promoting oxidative stress and atherosclerosis induced by Hcy.

## Materials and Methods

### Animals and treatment protocols

A total of 36 male C57BL/6J *ApoE* ^*–*/ *–*^ mice (6 weeks old; 25 g) were obtained from the Animal Center of Peking University Health Science Center (Beijing, China). They were kepted individually in a climate-controlled room at 24°C with 60% humidity. A total of 12 male C57BL/6J mice (6 weeks old; 20–25 g; Animal Center of Peking University Health Science Center) were fed with a regular mouse diet (Joint Feed Co., Ltd, Beijing, China) and used as the normal control group (N-control). The 36 C57BL/6J *ApoE* ^*–*/ *–*^ mice were divided into three groups ( *n*=12/group) and maintained for 15 weeks on the following diets: (1) *ApoE* ^*–*/ *–*^ mice control group (A-control), fed with a regular mouse diet; (2) Meth group, fed with a regular diet plus 1.7% methionine to establish a hyperhomocysteinemia (HHcy) model; (3) Meth-F group, fed with regular diet plus 1.7% methionine (wt/wt), 0.006% folate and 0.0004% vitamin B _12_ (Weitonglihua, Beijing, China). All mice had free access to food and water. The treatment of laboratory animals and the experimental protocol used followed the guidelines set out by the Ningxia Medical University (Yinchuan, China) and was approved by the Committee for Laboratory Animal Care of Ningxia Medical University. On the morning of the final day of the diet period, the mice in each group were anesthetized with 20% ethylcarbamate (1000 mg/kg) by intraperitoneal injection, blood was collected by cardiac puncture, and the mice were euthanized by exsanguination. The serum was separated by centrifugation (1000 *g* for 10 min at 4°C) and all samples were stored at –80°C prior to further analysis. The mice were grouped and treated as described in our previous study [17].

### THP-1-derived macrophage culture

THP-1, a human monocytic cell line, was obtained from the American Type Culture Collection (Manassas, USA) and the cells were grown in Roswell Park Memorial Institute (RPMI) 1640 medium (Thermo Fisher Scientific, Waltham, USA) supplemented with 15% fetal bovine serum (FBS; Thermo Fisher Scientific), 100 U/mL penicillin and 100 μg/mL streptomycin at 37°C in a 5% CO _2_ atmosphere, to a density of 4×10 ^6^ cells/mL. THP-1 cells were subsequently differentiated into macrophages by incubation with 500 μM phorbol myristate acetate at 37°C for 24 h. In the Hcy groups, cells were treated with 50, 100, 200 or 500 μM Hcy for 72 h. In the antagon group, cells were treated with 100 μM Hcy, 30 μM folate and 30 μM vitamin B _12_ for 72 h.

### Determination of serum Hcy and lipid levels in mice

The concentrations of Hcy, total cholesterol (TC), triglyceride (TG), high-density lipoprotein (HDL) and low-density lipoprotein (LDL) were detected with the ADVIA 2400 Chemistry System (Siemens AG, Munich, Germany) by conventional enzymatic methods [17].

### Tissue preparation and evaluation of AS lesions

Following cardiac puncture, the aorta was harvested, sampled, washed with distilled water, dipped in 78% methanol and stained for 40 min with 0.16% Oil-Red-O dissolved in 78% methanol/0.2 M NaOH (Merck KGaA) at room temperature. For morphological analysis, 7 μm frozen sections were stained with hematoxylin and eosin (H&E) according to standard protocols. For quantification of the lesion area, serial cross sections of the aortic roots (7 μm thick) and five serial sections were prepared from each location and conventionally stained with Oil-Red-O according to the method described by Yang *et al*. [17]. The lesion areas were quantified by a single observer blinded to the experimental protocol. The stained plaque areas were quantified using Image Pro Plus software version 4.5 (Media Cybernetics, Rockville, USA).

### Superoxide anion detection

To evaluate the O _2_^–^ production by the aorta and macrophages, the membrane permeable indicator dichlorofluores *cin* diacetate (H _2_DCF-DA) was used. The samples were combined with 10 μM H _2_DCF-DA (JianCheng Biotech, Nanjing, China) in serum-free Dulbecco’s modified Eagle’s medium (Thermo Fisher Scientific) at 37°C for 30 min and subsequently washed twice with PBS. Following pre-incubation at 37°C with 10 μM atorvastatin (Honghui Bio-Pharmaceutical, Beijing, China), N-acetyl-L-cysteine (NAC; 1 mM) or 4′,6-diamidino-2-phenylindole (DPI; 10 μM) was added and incubated for 30 min, the samples were analyzed with a flow cytometer (BD Biosciences, Franklin Lakes, USA) at an excitation wavelength of 488 nm and an emission wavelength of 525 nm. Superoxide anion concentration was determined by comparing the changes in fluorescence intensity using the BD Cytometric Bead Array (CBA) FCAP Array Software v3.0 (BD Biosciences).

### Reverse transcriptase-quantitative polymerase chain reaction (RT-qPCR)

Total RNA was extracted from 80 mg aorta using Trizol reagent (Thermo Fisher Scientific) according to the manufacturer’s protocol. RNA was reverse transcribed using a RevertAid First Strand cDNA Synthesis kit (Thermo Fisher Scientific) according to the manufacturer’s protocol. A SYBR Green kit (Thermo Fisher Scientific) was used for RT-qPCR analysis. The primer sequences are listed in Table 1. The reaction steps comprised of an initial activation step at 95°C for 10 min, followed by 45 cycles of 2-step PCR program of 95°C for 15 s and annealing temperatures ( Table 1) for 30 s, and finally 60°C for 30 s. An amplification curve was obtained for each qPCR run. The relative change in DNMT1 mRNA expression was determined by the fold change analysis (N) using the 2 ^–∆∆Cq^ method [18]. *GAPDH* was used as the internal reference.

**Table:** **Table 1** The primer sequences of *ECSOD* and *DNMT1* for real-time PCR analysis

Gene	GenBank No.	Sequence (5′→3′)	Length (bp)	Tm (°C)
*EC-SOD*	NM_011435.3	F: TGGATGCTGCCGAGATGC R: TGGATGGCACGGTTGGAG	181	53.0
*DNMT1*	NM_010066	F: GGAGCCCAGCAAAGAGTA R: GGGAGACACCAGCCAAAT	216	57.0
*GAPDH*	NM_008084.2	F: AAAGTGGAGATTGTT R: ATTTGATGTTAGTGG	181	45.4

F, forward; R, reverse; Tm, annealing temperature.

### EC-SOD promoter sequence analysis

Human EC-SOD gene 5′-flanking sequence was retrieved from the Eukaryotic Promoter Database ( epd.vital-it.ch). The core promoter of EC-SOD was analyzed by the Berkeley Drosophila Genome Project ( fruitfly.org:9005/seq_tools/promoter.html) and MethPrimer ( urogene.org/methprimer/index1.html).

### Promoter activity assay

The EC-SOD promoter reporter gene assay was performed using the Dual-Luciferase reporter system (Promega, Madison, USA) according to the manufacturer’s protocols. The EC-SOD 5′-flanking sequences were PCR-amplified from human macrophage genomic DNA using the primers listed in Table 2. DNA was extracted from macrophages, and PrimeSTAR Max DNA Polymerase (Cat No R045A; TaKaRa, Dalian, China) was used for PCR. Primers are listed in Table 2. Thermocycling conditions comprised a denaturation step at 94°C for 30 s, followed by 30 cycles of a primer annealing at 55°C for 30 s, and synthesis of DNA for 1 min at 72°C. These PCR products were fused to the firefly luciferase reporter vector pGL3-basic by *Eco*RI and *Han*dIII digestion. When the macrophages grew to 80% confluence in a 12-well plastic dish, they were transfected with 0.1 μg pGL3-basic or equimolar pGL3-ECSOD constructs using Lipofectamine 2000 reagent (Thermo Fisher Scientific) at 37°C for 24 h. The *Renilla* luciferase expression vector phRL-TK (0.005 μg; Promega), whose activity was unaffected by homocysteine, was cotransfected as the internal control. Cell lysates were prepared for the luciferase assay using luciferin and a luminometer (Promega). Promoter activities were normalized to phRL-TK and expressed as fold increase over pGL3-basic.

**Table:** **Table 2** Fragments of EC-SOD promoter region

Fragment	Primer sequence (5′→3′)	Length (bp)	Tm (°C)
2000	F: CCGCTCGAGCCTAGTAGCTGGGACTAC R: CCGCTCGAGCCTAGTAGCTGGGACTAC	1916	58
700	F: CCGCTCGAGGGCTGCTATGATTGAG R: GAAGATCTCGGGTTGTAGAATTGC	696	48
1100	F: CCGCTCGAGTTAGTTTGAGTTTTTCC R: GAAGATCTCGGGTTGTAGAATTGC	1317	45
1400	F: CCGCTCGAGTAGGATTTCAAGAACTGAAT R: GAAGATCTCGGACGTTGTAGAATTGCTT	1317	50
200	F: CCGCTCGAGTAGGATTTCAAGAACTG R: GGAAGATCTAATGTTTATTGGGTGCTC	200	60

F, forward; R, reverse; Tm, annealing temperature.

### EC-SOD methylation assay

Genomic DNA was isolated from the aorta and macrophages using a Wizard Genomic DNA Purification kit (Promega). An integrated DNA denaturation and bisulfite conversion process was accomplished in one step using an EZ DNA Methylation-Gold kit (Zymo Research, Irvine, USA). nMS-PCR consisted of two-step PCR amplification following a standard sodium bisulfite DNA modification. The first step used an outer primer pair that did not contain any CpG. The second-step PCR was carried out using the conventional PCR primers. The primers used and product sizes of the nMS-PCR assays are listed in Tables 3 and 4. To increase the efficiency, touchdown (TD) PCR was used in the amplification. Samples were subject to 30 cycles in a TD program (94°C for 30 s, 69°C for 30 s, and 72°C for 1 min, followed by a 0.5°C decrease in the annealing temperature every cycle; 20 cycles in total). After completion of the TD program, 20 cycles were run (94°C for 45 s, 54°C for 45 s and 72°C for 45 s), ending with a 5 min extension at 72°C. The PCR products were separated by electrophoresis on a 2% agarose gel containing ethidium bromide. The DNA bands were visualized by ultraviolet and quantified using a Gel Documentation and Analysis System ChemiDoc XRS system with Image Lab software (Version 4.1; Bio-Rad, Hercules, USA). Relative methylation level was calculated using the following formula: Methylation=methylation/(methylation+unmethylation)×100%.

**Table:** **Table 3** The nMS-PCR primers of EC-SOD for mice (NM_011435.3)

Primer set	Primer sequence (5′→3′)	Length (bp)	Tm (°C)
EC-SOD-O	F: TTTTTGAATAGAATGAAGAGGGTGTA R: AACCAAATCAAAATTTCAATCATAAA	543	70.4
EC-SOD-M	F: AGTAATGATGGAGAGGTTAGGTTTC R: AAATAAAACAAAAAAAACACTCGTA	110	64.5
EC-SOD-U	F: GTAATGATGGAGAGGTTAGGTTTTG R: AAAATAAAACAAAAAAAACACTCATA	110	64.1

O, outer primer; M, methylation primer; U, unmethylation primer; F, forward; R, reverse; Tm, annealing temperature.

**Table:** **Table 4** The nMS-PCR primers of EC-SOD for macrophage (NM_003102.2)

Primer set	Primer sequence (5′→3′)	Length (bp)	Tm (°C)
EC-SOD-O	F: TTAGTTTTTTTAGTAGTTGGGATTATAGGT R: CACAACAATTAAACAATTCACAATAAC	286	58.0
EC-SOD-M	F: GTTTAGGTTGGAGTGTAGTGGC R: AATCGAAATCATCCTAACTAACACG	186	57.8
EC-SOD-U	F: GTTTAGGTTGGAGTGTAGTGGTGT R: TCAAAATCATCCTAACTAACACAAT	184	56.1

O, outer primer; M, methylation primer; U, unmethylation primer ; F, forward; R, reverse; Tm, annealing temperature.

### Determination of aortic SAM and SAH concentrations

SAM and SAH concentrations were determined by high performance liquid chromatography (HPLC). Briefly, the aortic tissues (20 mg) were homogenized, mixed thoroughly with 1 mL of 20% HClO _4_ solution, and samples were loaded onto a C18 column (Shimadzu Corporation, Kyoto, Japan) and run by a Hitachi L2000 HPLC system (Hitachi, Tokyo, Japan). SAM and SAH standards were used to identify the elution peaks, and chormatograms were recorded and the SAM and SAH values of the tissues were calculated based on the standard curve [18].

### Gene recombination and siRNA interference

DNA was extracted from macrophages and PrimeSTAR Max DNA polymerase (Cat No R045A; TaKaRa) was used for PCR. Primers are listed in Table 5. Thermocycling conditions comprised a denaturation step at 94°C for 30 s, followed by 30 cycles of a primer annealing step at 58°C for 30 s, and synthesis of DNA for 1 min at 72°C. The plasmid was cleaved by the restriction enzymes and the target gene fragments were ligated by T4 DNA ligase (Thermo Fisher Scientific) and transfected into *Escherichia coli* DH5α cells (TaKaRa) by heat-shock at 42°C for 30 s. The transfected bacterial cells were incubated at 37°C overnight with disks to allow clone formation. Following incubation, single clones were isolated and inoculated into Luria-Bertani medium (Thermo Fisher Scientific). The fragments were identified by electrophoresis on a 1% agarose gel. Following identification, the positive fragments were subject to sequencing. The correct fragments were ligated with pcDNA3.1 (Hanbio Biotechnology, Shanghai, China) and transformed into bacteria from which individual clones were expanded. The plasmid DNA was subject to digestion with *Eco*RI and *Hin*dIII, and positive clones were confirmed by 2% gel electrophoresis. The recombinant plasmid pcDNA3.1-EC-SOD/DNMT1 fusion gene was then established. Prior to transfection, the cells were grown to 80% confluence. Complexes of pcDNA3.1-EC-SOD/DNMT1 and 1 μL Lipofectamine 2000 were prepared and added to each well of a 96-well plate. The fluorescence intensity was used to identify the transfection efficiency using a phase contrast inverted fluorescence microscope (Olympus IX71; Olympus, Tokyo, Japan; Magnification, ×100). Twenty-four hours later, the activity of EC-SOD/DNMT1 was detected by PCR. Western blot analysis was performed to examine the fusion gene expression.

**Table:** **Table 5** Primer sequences for *EC-SOD* gene recombination

Gene	GenBank No.	Primer sequence (5′→3′)	Length (bp)	Tm (°C)
*EC-SOD*	NM_003102.2	F: CCCAAGCTTATGCTGGCGCTACTGTGTTC R: CGGAATTCTCAGGCGGCCTTGCACTCG	718	58.0
*DNMT1*	NM_001379.2	F: ATTCGCTAGCACCGGCGCGTACCGCCCC R: ATTCGAATTCGCCTTAGCAGCTTCCTCCT	5185	52.0

F, forward; R, reverse; Tm, annealing temperature.

siRNAs of EC-SOD were supplied by Thermo Fisher Scientific and sequences are shown in Table 6. Macrophages were transfected with siRNAs when they reached 80% confluence. For each sample, complexes of siRNA molecule-Lipofectamine 2000 were prepared and added to 96-well plates. Detection of fluorescence intensity was performed with a fluorescence microscope to identify the transfection efficiency. The transfected macrophages were treated with 100 μM Hcy at 37°C for 24 h and then subject to qPCR analysis. Western blot analysis was performed to examine the fusion gene expression using antibodies against EC-SOD (Cat No. sc-271170; Santa Cruz Biotechnology, Dallas, USA).

**Table:** **Table 6** The siRNA sequences for EC-SOD

Fragment	Sequences of siRNAs (5′→3′)
474(S1)	F: GCGCCAAGCUCGACGCUUTT R: AAGGCGUCGAGCUUGGCGCTT
1155(S1)	F: GGCCUCCAUUUGUACCGAATT R: UUCGGUACAAAUGGAGGCCTT
1266(S3)	F: CCUUUGACCUGACGAUCUUTT R: AAGAUCGUCAGGUCAAAGGTT

F, forward; R, reverse.

### Western blot analysis

Total proteins were isolated from the cells using cell lysis buffer (Keygen Biotech, Nanjing, China) and protein concentrations were quantified using a Bicinchoninic Acid Protein Assay kit (Millipore, Billerica, USA). Equal amounts of protein (80 μg) were separated by 12% SDS-PAGE and transferred onto polyvinylidene fluoride membranes (Millipore). Membranes were subsequently blocked in 5% skimmed milk for 2 h at room temperature. Membranes were incubated with antibodies against EC-SOD (1:500) and β-actin (Cat No. sc-70319; 1:2000; Santa Cruz Biotechnology) at 4°C overnight, followed by incubation with horseradish peroxidase (HRP)-conjugated goat anti-mouse IgG secondary antibody (Cat No. sc-2031; 1:2000; Santa Cruz Biotechnology) for 2 h at room temperature. The protein bands were visualized and analyzed using a Gel Documentation and Analysis System (ChemiDoc XRS System; Bio-Rad) with Image Lab software (version 4.1).

### Statistical analysis

Data are expressed as the mean±SD. The data were analyzed using one-way analysis of variance and additional analysis was done using the Student-Newman-Keuls test for multiple comparisons within groups or a Student’s *t*-test for the comparison of two groups. Correlation analysis was performed using Pearson’s correlation analysis. *P*<0.05 was considered to be statistically significant.

## Results

### Elevated superoxide anion promotes the formation of atherogenic lesions induced by HHcy

**Figure 1:**
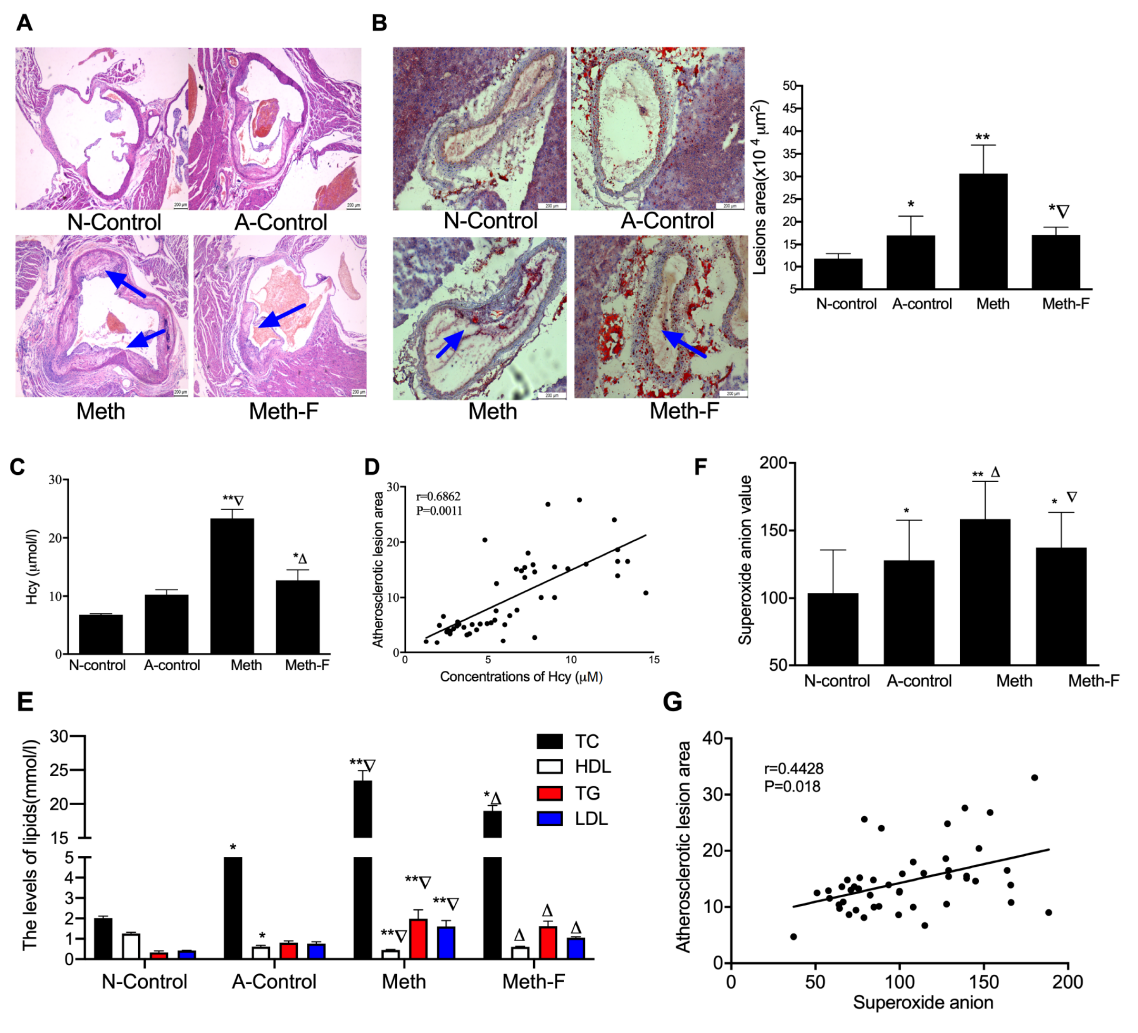
Figure 1 Hcy promotes formation of atherogenic lesions in *ApoE* ^–/–^ mice via superoxide anion (A) Hematoxylin and eosin staining of representative sections from the aorta of the ApoE ‒/‒ mice fed with high-methionine diet for 15 weeks. The arrows in the images indicate the presence of atherogenic lesions. (B) Oil red O staining of representative sections from the aortic root, arrows in the images indicate the presence of atherogenic lesions. Magnification, ×100. Right penal: relative AS plaque areas of the total cross-sectional vessel wall area. (C,D) Serum levels of Hcy in ApoE ‒/‒ mice and the correlation between plaque area and serum levels of Hcy evaluated by Pearson correlation analysis. (E) The levels of lipid markers (TC, TG LDL and HDL) in the serum of ApoE ‒/‒ mice. (F) The superoxide anion content in ApoE ‒/‒ mice. (G) The correlation between the superoxide anion and plaque area from ApoE ‒/‒ mice were calculated by Pearson’s correlation analysis. * P<0.05 and ** P<0.01 vs the N-control group; ▽ P<0.01 vs the A-control group; △ P<0.01 vs the Meth group. Hcy, total homocysteine; LDL, low-density lipoprotein; TG, triglyceride; HDL, high-density lipoprotein; Hcy, homocysteine; TC, total cholesterol.

To evaluate the atherogenic effects of HHcy in *ApoE* ^–/–^ mice fed with high-methionine diet, the atherogenic lesions were examined by H&E ( Figure 1A) and Oil Red O staining ( Figure 1B). Results showed that the area of atherogenic lesions was significantly increased in *ApoE* ^–/–^ mice fed with high-methionine diet ( Figure 1B), while the area of atherogenic lesion was decreased in *ApoE* ^–/–^ mice after the mice were fed with high-methionine and folate and vitamin B _12_ diet. The concentration of Hcy was increased in Meth group, and decreased when *ApoE* ^–/–^ mice were fed with high-methionine and folate and vitamin B _12_ ( Figure 1C). In addition, it was also found that the Hcy concentration in serum was positively correlated with the atherosclerotic lesion areas (r=0.6862; *P*=0.0016; Figure 1D). Furthermore, TG, TC and LDL levels were increased in *ApoE* ^–/–^ mice fed with a high-methionine diet, whereas the contents of lipids were suppressed when the mice were feed with folate and vitamin B _12_ ( Figure 1E). However, HDL concentrations were decreased in the high-methionine diet groups and increased after mice were fed with folate and vitamin B _12_ diet ( Figure 1E), implying that a high methionine diet may induce HHcy in *ApoE* ^–/–^ mice, while folate and vitamin B _12_ are able to ameliorate the effects of HHcy.

Oxidative stress plays a critical role in the occurrence and development of AS, and reactive oxygen species (ROS) and their reaction products are important mediators in vascular pathology and oxidative stress, particularly superoxide anions [22]. After *ApoE* ^–/–^ mice were fed with high-methionine diet, the levels superoxide anions were increased ( Figure 1F). The atherogenic lesion areas were positively correlated with the levels of superoxide anions (r=0.4428; *P*=0.018; Figure 1G), indicating that superoxide anions may enhance the development of atherogenic lesions.

### EC-SOD expression is suppressed by Hcy *in vivo* and *in vitro*

**Figure 2:**
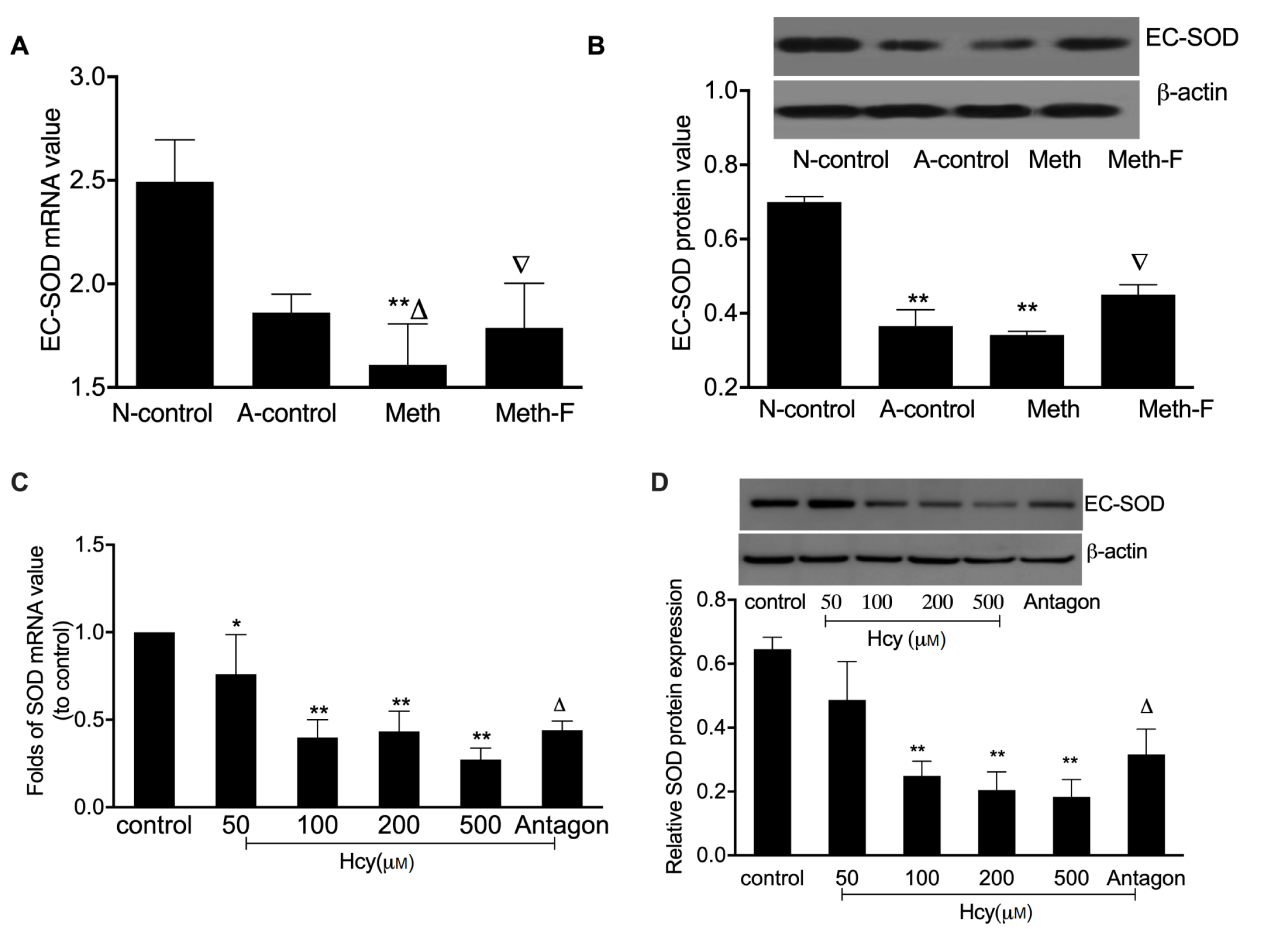
Figure 2 EC-SOD is suppressed by Hcy in aorta and macrophages (A) Following 15 weeks of treatment, the level of EC-SOD mRNA in the aorta of ApoE ‒/‒ mice was detected by qRT-PCR. (B) Western blot analysis of EC-SOD expression in aorta of ApoE ‒/‒ mice. (C) qRT-PCR was used to measure the expression of EC-SOD mRNA levels in macrophages after the cells were treated with different concentrations of Hcy for 24 h. (D) The EC-SOD protein expression was measured by western blot analysis. * P<0.05 and ** P<0.001 vs the control group; ▽ P<0.01 vs the A-control group; △ P<0.01 vs the Meth group in the (A,B). △ P<0.05 vs the 100 μM Hcy group in the (C,D). Antagon: 100 μM Hcy and 30 μM vitamin B 12 and 30 μM folate acid.

It has been reported that EC-SOD is constituted as an important defense mechanism against superoxide anions in the arterial microenvironment [23]. To confirm the effect of Hcy on EC-SOD expression *in vivo* and *in vitro*, the expressions of EC-SOD were detected in the aorta and macrophage. Our results showed that EC-SOD mRNA levels were decreased in *ApoE* ^–/–^ mice fed with high-methionine, and were increased when the *ApoE* ^–/–^ mice were fed with folate and vitamin B _12_ ( Figure 2A). Consistently, the protein levels of EC-SOD in aorta were decreased when the *ApoE* ^–/–^ mice were fed with high-methionine, and increased after the mice were fed with folate and vitamin B _12_ ( Figure 2B). In line with the expression of EC-SOD *in vivo*, EC-SOD expression levels were suppressed in macrophages after the cells were treated with various concentrations of Hcy ( Figure 2C,D). These findings provided ample evidence that EC-SOD may be associated with the macrophage response to oxidative stress, and folate and vitamin B _12_ may protect the cells from oxidative damage caused by the toxicity of Hcy.

### EC-SOD plays a major role in the resistance to oxidative stress in macrophages induced by Hcy

**Figure 3:**
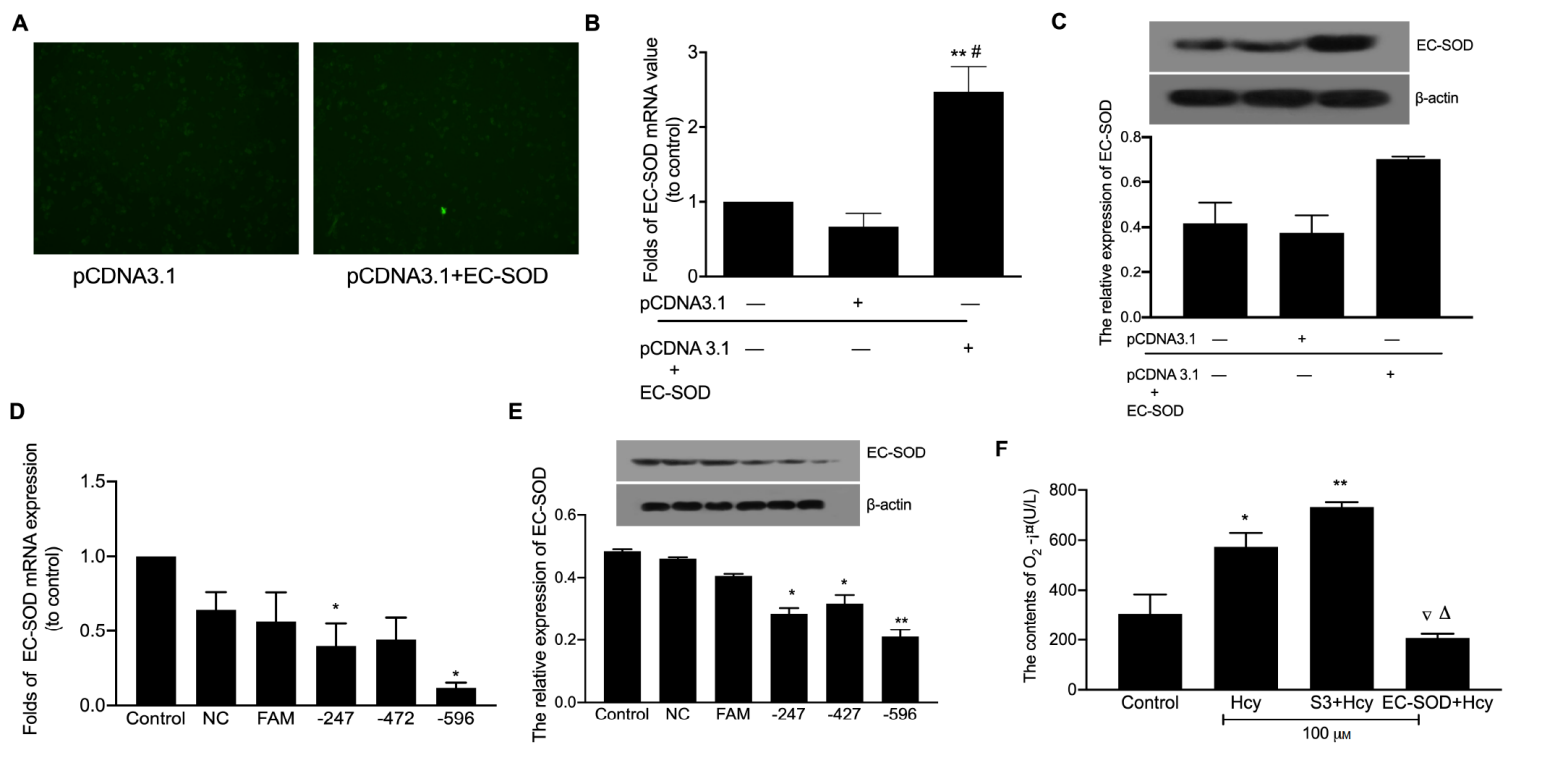
Figure 3 Effect of overexpression and downregulation of EC-SOD in macrophages (A) The control group and cells transfected with pCDNA3.1+EC-SOD were analyzed by fluorescence microscopy. Magnification, ×100. Following transfection of macrophages with recombination vectors of EC-SOD, (B) EC-SOD mRNA expression was analyzed by RT-PCR and (C) EC-SOD protein expression was measured by western blot analysis. Following transfection of macrophages with different RNA inference fragments, (D) EC-SOD mRNA expression was measured by qRT-PCR and (E) EC-SOD protein expression was detected by western blot analysis. (F) The contents of O 2 –· was detected by flow cytometry following the over-expression or silencing of EC-SOD in macrophages treated with 100 μM Hcy. * P<0.05 and ** P<0.01 vs untreated cells; # P<0.05 vs the pCDNA3.1 group; △ P<0.05 vs the S3+Hcy group; ▽ P<0.05 vs 100 μM Hcy group. NC, negative control; FAM, labeled fluorescent of 6-carboxy-fluorescein; pCDNA3.1, vector with enhanced green fluorescent protein; pCDNA3.1-ECSOD, recombinant plasmid of EC-SOD; S3+Hcy group, macrophages transfected with the ‒596 fragment and treated with 100 μM Hcy; EC-SOD+Hcy group, macrophages transfected with the recombination vector and treated with 100 μM Hcy.

To verify the role of EC-SOD in atherosclerosis via oxidative stress induced by Hcy, we overexpressed EC-SOD or knocked down *EC-SOD* to confirm the role of EC-SOD in Hcy-induced oxidative stress in macrophages. Our observation of green fluorescence verified that the recombined vector was successfully transfected into the macrophages ( Figure 3A). Subsequently, EC-SOD mRNA levels were significantly increased in the pcDNA3.1-ECSOD transfection group ( Figure 3B). EC-SOD protein level was significantly increased in the pcDNA3.1-ECSOD group ( Figure 3C). Furthermore, the expression of EC-SOD mRNA was significantly decreased after cells were transfected with the –247 and –596 fragments ( Figure 3D). Meanwhile, western blot analysis demonstrated that the EC-SOD protein level was decreased ( Figure 3E). These results indicated that the –596 fragment was the most effective RNA interference fragment.

The superoxide anion content was measured following transfection with the recombined vector and the –596 RNA interference fragment for EC-SOD ( Figure 3F). Superoxide anion content was significantly increased in macrophages after treatment with Hcy. Conversely, the superoxide anion content was significantly decreased with EC-SOD overexpression and Hcy treatment ( *P*<0.05). Taken together, these results suggest that EC-SOD plays a major role in the oxidative stress in macrophages induced by Hcy.

### EC-SOD hypomethylation in the aorta of *ApoE* ^–/–^ mice induced by Hcy

**Figure 4:**
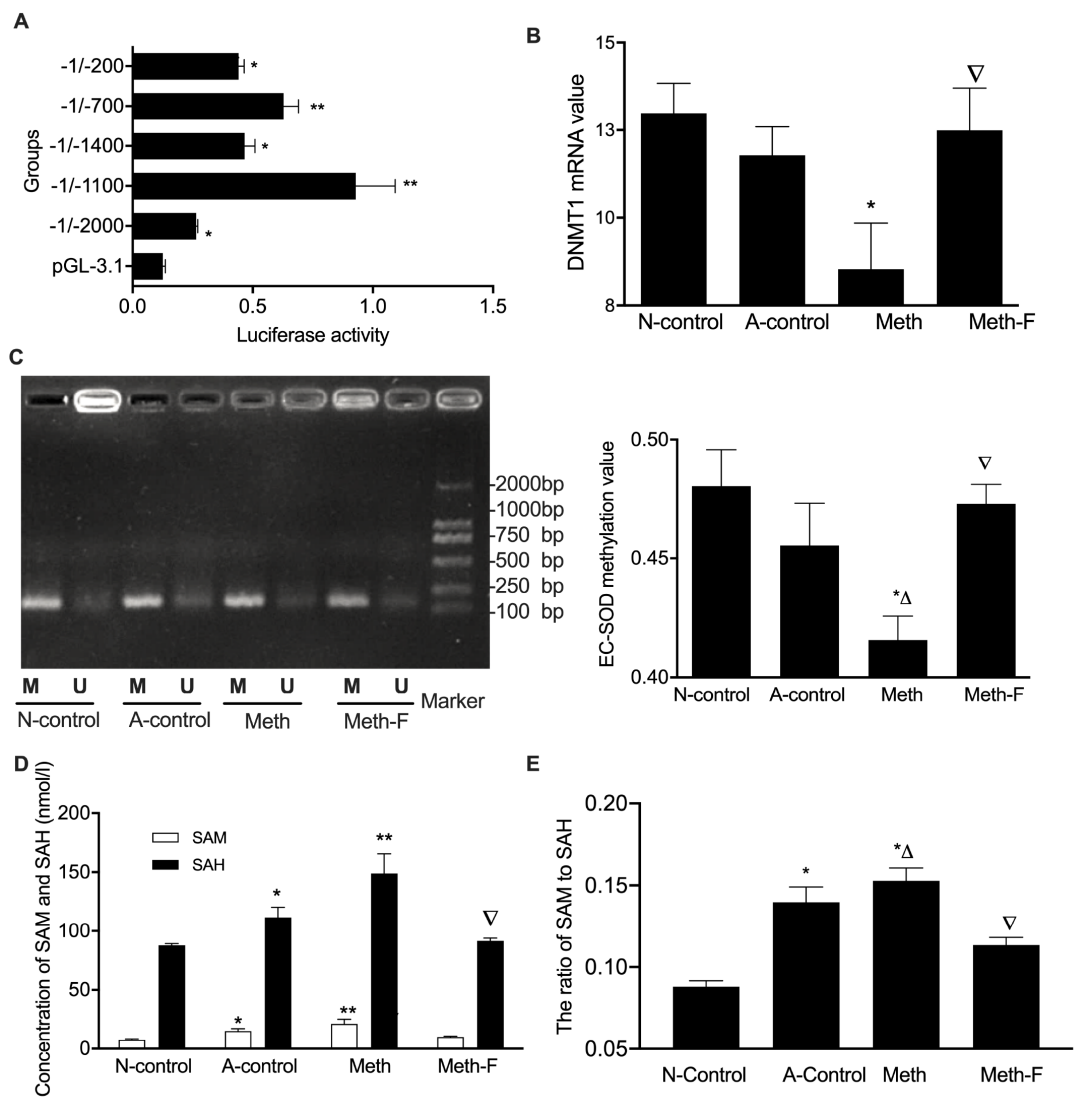
Figure 4 Hcy suppresses EC-SOD methylation levels in the aortas of *ApoE* ^–/–^ mice (A) Analysis of EC-SOD promoter activity. Different lengths of human EC-SOD 5′-flanking sequences were fused to the luciferase gene in a pGL-3.1. The fragments of promoter activity were subsequently analyzed by calculating the ratio of luminescence from the experimental reporter vector to the luminescence from a control vector (phRL-TK). (B) The expression of DNMT1 mRNA in the aorta of mice was detected by qRT-PCR. (C) The methylation status of EC-SOD in the aortas of mice was analyzed by methylation special touch down polymerase chain reaction. (D) The concentrations of SAH and SAM in the aorta of mice were measured by HLPC. (E) The ratio of SAM to SAH. * P<0.05 and ** P<0.01 vs the N-control group or the pGL3-basic group; △ P<0.05 vs the A-control group; ▽ P<0.05 vs the Meth group. pGL-3.1, pGL3-basic vector; M, methylated polymerase chain reaction band; U, unmethylated polymerase chain reaction band; SAM, S-adenosylmethionine; SAH, S-adenosylhomocysteine.

Transcription activity is the key to determine gene expression levels in eukaryotic organisms, which is susceptible to various factors such as epigenetic modification including DNA methylation [24]. To get insight into the underlying mechanism of EC-SOD in the formation of atherosclerotic plaque, we transfected several fragments of EC-SOD 5′-flanking region (–200/–1, –700/–1, –1100/–1, –1400/–1 and –2000/–1) that were inserted into the firefly luciferase vector pGL3. Luciferase activity assay revealed that the region (–1100/–1) which spans most of the CpG dinucleotide of EC-SOD promoter region has the highest promoter activity ( Figure 4A), indicating that the regulatory elements for EC-SOD transcription possibly exist in this region. In our previous study, we confirmed that DNMT1 plays an important role in the formation of atherosclerotic plaque [25]. In the present study, we found DNMT1 mRNA expression was suppressed in *ApoE* ^–/–^ mice ( Figure 4B). The DNA methylation pattern has been considered to be a useful molecular marker of AS due to its role in silencing DNA transcription [26]. DNA methylation of EC-SOD was analyzed to explore the cause of EC-SOD expression changes. It was found that EC-SOD methylation was suppressed in the Meth group, and moderated after *ApoE* ^–/–^ mice were fed with folate and vitamin B _12_ ( Figure 4C), which indicated that Hcy suppressed EC-SOD DNA methylation.

SAM is an important intermediate in the transmethylation reaction, while SAH is a potent inhibitor of cellular transmethylation [27]. In our previous study, we confirmed that the ratio of SAM to SAH is a more sensitive indicator for the diagnosis of AS [28], but the role of this ratio in gene’s promoter methylation is still unknown. To illustrate the mechanism of HHcy-mediated EC-SOD DNA methylation in *ApoE* ^–/–^ mice, we analyzed SAM and SAH levels by HPLC. It was found that the concentrations of SAM and SAH were significantly increased in *ApoE* ^–/–^ mice fed with high-methionine ( Figure 4D). As predicted, the ratio of SAM to SAH was promoted in *ApoE* ^–/–^ mice fed with high-methionine ( Figure 4E), suggesting that the efficiency of transfer of methyl groups from SAM to the numerous methyl acceptors is significantly decreased, and it may be a crucial reason for EC-SOD hypomethylation.

### DNMT1 plays a key role in EC-SOD hypomethylation in macrophages

**Figure 5:**
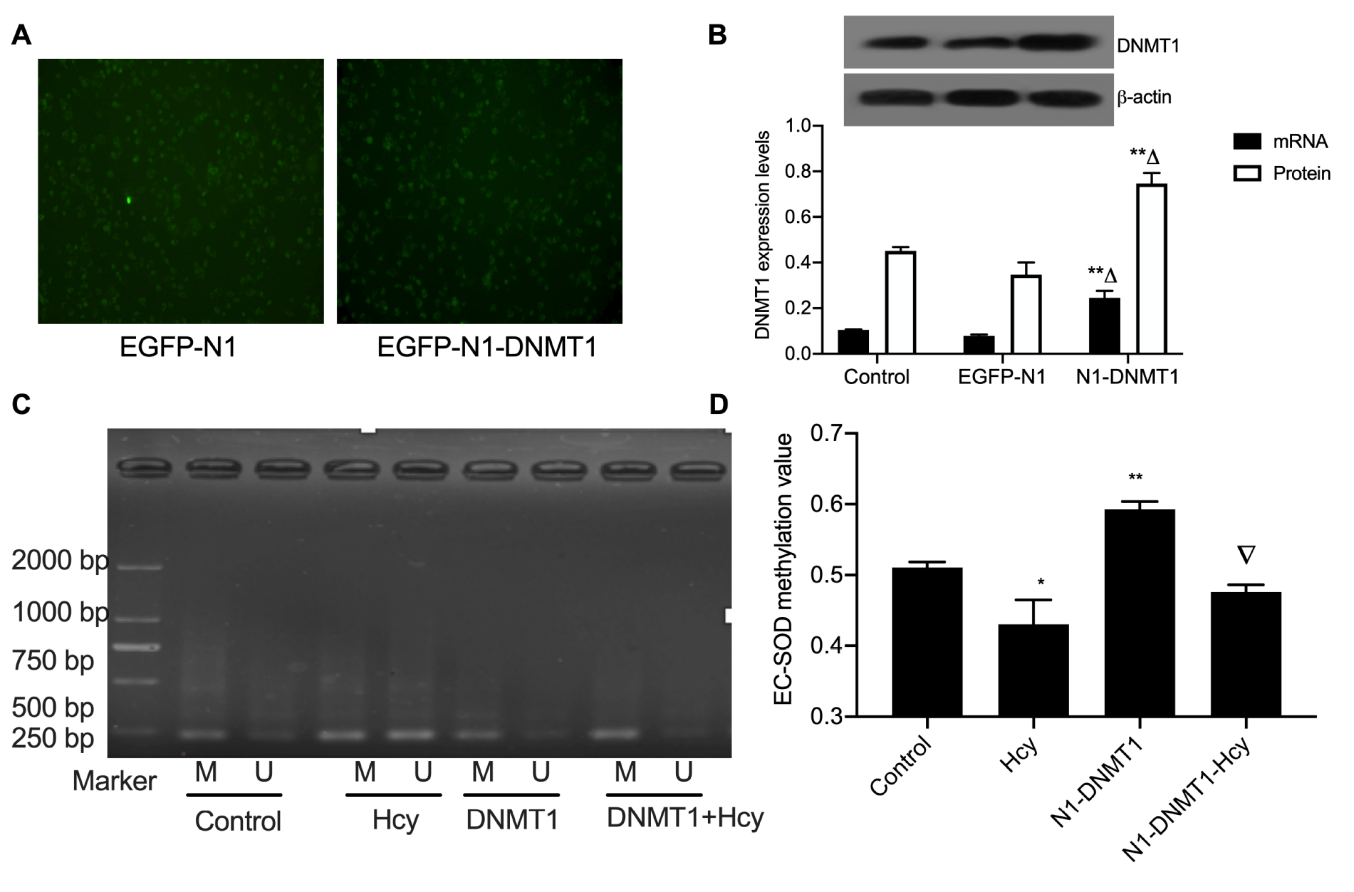
Figure 5 Expression of EGFP-N1-DNMT1 plasmid transfected into macrophages (A) The EGFP-N1-DNMT1 plasmid was transfected into macrophages and the transfection efficiency was detected with a fluorescence microscope and analyzed using Image pro Plus software. Magnification, ×100. (B) The level of DNMT1 mRNA was measured by qRT-PCR and the DNMT1 protein expression was measured by western blot analysis. (C) pEGFP-N1-DNMT1 was transfected into macrophages for 24 h, and then cells were treated with 100 μM Hcy in complete medium for another 24 h. EC-SOD methylation was detected. * P<0.05 and ** P<0.01 vs the control group; △ P<0.05 vs the pEGFP-N1 group; ▽ P<0.05 vs the N1-DNMT1 group. Control, cells not transfected that were cultured in complete medium; Hcy, macrophages treated with 100 μM Hcy; N1-DNMT1, macrophages transfected with EGFP-N1-DNMT1 then treated with complete medium; N1-DNMT1-Hcy, macrophages transfected with EGFP-N1-DNMT1 then treated with 100 μM Hcy. EGFP, enhanced green fluorescent protein.

We previously found that DNMT1 plays an important role in the regulation of ABCA1 and ACAT1 methylation in THP-1 monocyte-derived foam cells induced by Hcy [29]. To validate whether DNMT1 participates in the transcriptional regulation of EC-SOD, the recombination vector of DNMT1 was transfected into macrophages, and our results verified that the recombination vector was successfully transfected into the macrophages ( Figure 5A). Subsequently, the mRNA and protein levels of DNMT1 were detected by qPCR and western blot analysis respectively ( Figure 5B). DNMT1 expression levels were significantly increased following recombination with N1-DNMT1. The impact of over-expression of DNMT1 on the EC-SOD methylation level was also investigated ( Figure 5C). EC-SOD methylation level was significantly increased by 1.6 folds in the EGFP-N1-DNMT1 group compared with that in the control group ( Figure 5D). These results support the hypothesis that Hcy-induced DNMT1 inhibition mediates the demethylation of the EC-SOD promoter.

## Discussion

Our previous study suggested that increased concentration of plasma Hcy accelerates the progression of AS and promotes atherothrombotic events [30], and oxidative stress was also found to be increased in the AS vessel walls in patients exhibiting HHcy [28], but the precise underlying mechanisms are still unknown. In the present study, we revealed that the activity of superoxide anion was increased in the aorta in *ApoE* ^–/–^ mice fed with a high methionine diet. We also found that imbalance of oxidative stress existed in *ApoE* ^–/–^ mice induced by Hcy.

Oxidative stress is regulated by a variety of redox enzymes and antioxidants [29]. Antioxidants occur naturally within mammalian tissues where they protect against the harmful side effects of ROS by counteracting free radical reactions [31]. At present, the antioxidant enzyme EC-SOD is considered to be responsible for defending the body against oxygen free radicals, eliminating superoxide anions by converting them to O _2_ and hydrogen peroxide [32]. The aim of the present study is to demonstrate whether EC-SOD is a vital factor in the body’s defense against the superoxide anion. We found that EC-SOD expression was increased in the AS vessels of *ApoE* ^–/–^ mice and in macrophages treated with Hcy. The concentration of superoxide anion was decreased following transfection with pCDNA3.1-ECSOD, whereas the superoxide anion was increased when EC-SOD siRNA fragments were transfected into macrophages. This suggests that EC-SOD is a key factor for eliminating ROS.

DNA methylation is an important epigenetic mechanism that selectively regulates gene expression and is associated with cardiovascular diseases. Previous studies have revealed that specific changes in the DNA methylation pattern occur prior to the appearance of vascular lesions in *ApoE* ^–/–^ mice, particularly in the aorta and circulating inflammatory cells, including macrophages [33]. It has been reported that DNA hypomethylation is associated with changes in gene expression during the development of AS lesions [34]. In the present study, we observed that the -1/-1100 fragment with a larger number of CpG islands in the EC-SOD promoter region had the highest activity. CpG islands are potential sites of methylation and this may explain the regular occurrence of mutations and the methylation status of each of the CpG dimers located within the coding sequence as characterized by direct genomic sequencing. A previous study revealed that the number of methylated CpG dinucleotides was remarkably reduced in the EC-SOD gene in AS aortas compared with that in normal aortic intima-media [35]. A number of previous studies have reported that Hcy is regulated by the expressions of DNMTs, particularly DNMT1 [ 13, 36] . In the process of methyl transfer reactions, methionine is converted to Hcy with SAM and SAH as byproducts. As the only methyl group donor, SAM is converted to SAH, which is catalyzed by DNMT1 [37]. It was also revealed that Hcy promotes the expression of DNMT1 via hypomethylation of genes associated with AS [18]. In the present study, DNMT1 expression was found to be decreased in macrophage cells treated with Hcy and in the AS lesions of *ApoE* ^–/–^ mice, in accordance with the hypomethylation of EC-SOD.

As SAM is the major methyl group donor, while SAH is a potent inhibitor of the cellular transmethylation reaction, a reasonable deduction is that hypomethylation is a passive process due to the increase of SAM and the SAM/SAH ratio, as well as the decline of SAH [38]. As Hcy suppresses the expression of DNMT1, the transmethylation reaction rate is decreased and the concentrations of SAM and SAH, as well as the ratio of SAM/SAH, are increased. In the present study, we found that there was an increase in the concentration of SAM and in the ratio of SAM/SAH in the aorta of *ApoE* ^–/–^ mice. This observation is consistent with the hypothesis that SAM and SAH are key components in the pathophysiology of the Hcy-vascular disease axis.

The present study demonstrates that vitamin B _12_ and folate may relieve the effect of methylation in cultured macrophages treated with Hcy. The re-methylation process requires two key enzymes, i.e., methionine synthase with methylcobalamin as a coenzyme and methylene tetrahydrofolate reductase with 5-methyltetrahydrofolate as the methyl donor [ 39, 40] . Therefore, the addition of vitamin B _12_ and folate may influence the metabolism of Hcy and disturb the methylation and expression of EC-SOD.

In conclusion, the results of the present study indicate that Hcy may induce DNMT1 downregulation and EC-SOD DNA hypomethylation, which may be the major mechanism by which Hcy induces AS and may represent the important novel mechanisms by which Hcy induces an imbalance of oxidative and anti-oxidative effects. Additionally, an increased folate and vitamin B _12_ supplementation may be beneficial for the prevention of atherogenic diseases.
